# Towards biomarker-based optimization of deep brain stimulation in Parkinson’s disease patients

**DOI:** 10.3389/fnins.2022.1091781

**Published:** 2023-01-11

**Authors:** Jana Peeters, Alexandra Boogers, Tine Van Bogaert, Till Anselm Dembek, Robin Gransier, Jan Wouters, Wim Vandenberghe, Philippe De Vloo, Bart Nuttin, Myles Mc Laughlin

**Affiliations:** ^1^Experimental Oto-Rhino-Laryngology, Department of Neurosciences, KU Leuven, Leuven, Belgium; ^2^Department of Neurology, University Hospitals Leuven, Leuven, Belgium; ^3^Faculty of Medicine, University of Cologne, Cologne, Germany; ^4^Laboratory for Parkinson Research, Department of Neurosciences, KU Leuven, Leuven, Belgium; ^5^Experimental Neurosurgery and Neuroanatomy, Department of Neurosciences, KU Leuven, Leuven, Belgium; ^6^Department of Neurosurgery, University Hospitals Leuven, Leuven, Belgium

**Keywords:** evoked potentials, deep brain stimulation, electroencephalography, Parkinson’s disease, programming

## Abstract

**Background:**

Subthalamic deep brain stimulation (DBS) is an established therapy to treat Parkinson’s disease (PD). To maximize therapeutic outcome, optimal DBS settings must be carefully selected for each patient. Unfortunately, this is not always achieved because of: (1) increased technological complexity of DBS devices, (2) time restraints, or lack of expertise, and (3) delayed therapeutic response of some symptoms. Biomarkers to accurately predict the most effective stimulation settings for each patient could streamline this process and improve DBS outcomes.

**Objective:**

To investigate the use of evoked potentials (EPs) to predict clinical outcomes in PD patients with DBS.

**Methods:**

In ten patients (12 hemispheres), a monopolar review was performed by systematically stimulating on each DBS contact and measuring the therapeutic window. Standard imaging data were collected. EEG-based EPs were then recorded in response to stimulation at 10 Hz for 50 s on each DBS-contact. Linear mixed models were used to assess how well both EPs and image-derived information predicted the clinical data.

**Results:**

Evoked potential peaks at 3 ms (P3) and at 10 ms (P10) were observed in nine and eleven hemispheres, respectively. Clinical data were well predicted using either P3 or P10. A separate model showed that the image-derived information also predicted clinical data with similar accuracy. Combining both EPs and image-derived information in one model yielded the highest predictive value.

**Conclusion:**

Evoked potentials can accurately predict clinical DBS responses. Combining EPs with imaging data further improves this prediction. Future refinement of this approach may streamline DBS programming, thereby improving therapeutic outcomes.

**Clinical trial registration:**

ClinicalTrials.gov, identifier NCT04658641.

## 1. Introduction

Subthalamic nucleus (STN) deep brain stimulation (DBS) is an established neurosurgical therapy for advanced Parkinson’s disease (PD), involving implantation of a lead to precisely deliver electrical stimulation to the brain (Benabid et al., 1991; Limousin et al., 1998; Coenen et al., 2008; Kalia et al., 2013). Identification of optimal DBS settings is essential to maximize therapeutic outcome. However, even with accurate lead positioning this remains time-consuming and is highly dependent on programmer expertise (Volkmann et al., 2002; Picillo et al., 2016; Lange et al., 2021). Traditionally, DBS settings are selected *via* a monopolar review assessment, where the optimal DBS-contacts are identified by systematically evaluating the clinical response elicited when stimulating on each contact separately. With the advent of new technologies such as directional leads and multiple independent current-controlled (MICC) stimulators, the programming parameter space has expanded exponentially. This technology allows improved stimulation precision, and thus optimized DBS therapy, but comes at the cost of greatly increased programming complexity and time (Wagle Shukla et al., 2017; Santaniello et al., 2018; Koeglsperger et al., 2019). Finally, not all symptoms respond immediately to DBS meaning that a clinician may not be able to determine the best setting during a single clinical visit (Wagle Shukla et al., 2017). Consequently, not all DBS patients receive optimal therapy.

Imaging offers one potential solution to guide programming. This approach involves visualization of the lead and different contacts in relation to the relevant nuclei. Studies have shown that image-guided programming can be significantly less time-consuming whilst still leading to non-inferior motor improvements compared to conventional programming (Pourfar et al., 2015; Lange et al., 2021; Malekmohammadi et al., 2022). More recently, image-guided approaches also visualize the DBS-induced spread of electrical stimulation to give the programmer a clearer theoretical indication of the stimulated area, such as the electric field (EF), to guide and improve stimulation effects (Hemm et al., 2005; Åström et al., 2009; Nguyen et al., 2019). The EF overlap with the relevant image-derived anatomical structures can then be used to explain the DBS effects in an individual patient (Maks et al., 2009; Aström et al., 2010; Frankemolle et al., 2010; Mikos et al., 2011). Furthermore, individual patient data can be pooled to create probabilistic stimulation maps for different (clinical) outcome parameters (Butson et al., 2011; Akram et al., 2017; Gourisankar et al., 2018). For example, a recent study reported the reconstruction of probabilistic stimulation maps in PD patients covering the dorsolateral STN as well as surrounding white matter and predictive of a good motor outcome, a so-called sweet spot (Dembek et al., 2019), which has already been proposed to use as a programming guide (Phibbs et al., 2014). A recent functional magnetic resonance imaging (fMRI) study showed that clinically optimal stimulation produces a characteristic fMRI brain response marked by preferential motor circuit engagement, which is indicative of a functional sweet spot and hence, could be used as a biomarker of clinical response (Boutet et al., 2021). However, the precise location (and even existence) of a neuroanatomical sweet spot for DBS within STN remains disputed (Hamani et al., 2017).

Another potential solution is the development of electrophysiological biomarkers that can link DBS settings to patient-specific clinical outcomes. Such biomarkers may streamline the DBS programming process and have the potential to improve therapeutic outcomes in sub-optimally programmed patients. Intraoperative recordings of subthalamic local field potentials (LFP) are already being used to determine the final lead position but also to guide and improve DBS programming as reported by multiple research groups (Chen et al., 2022; Darcy et al., 2022; Hirschmann et al., 2022; Shah et al., 2022). Other studies have recorded cortical evoked potentials (EPs) in PD patients using both electroencephalography (EEG) (Walker et al., 2012) and electrocorticography (Miocinovic et al., 2018) approaches. These studies indicated that a short-latency EP peak around 3 ms may be useful in predicting clinical outcomes. Recently, a review article combined relevant DBS EP studies together and concluded that EPs may be useful as a biomarker for DBS parameter selection, especially with the expansion of the stimulation parameter space (Dale et al., 2022).

In a recent study (Peeters et al., 2021) we recorded similar short-latency EP at 3 ms (P3), in addition to a long-latency peak around 10 ms (P10) using EEG and concluded that changing the stimulation contacts significantly affected P3 and P10 peak amplitudes. In a follow-up study, we found that the peak amplitude was also significantly affected when applying MICC technology to change the stimulation depth (Peeters et al., 2022). Furthermore, by examining the correlation between EPs and imaging-derived information we found that P3 was largest when stimulating through the DBS-contacts closest to dorsolateral STN, while P10 was largest when stimulating through the DBS-contacts closest to substantia nigra (SN). This data indicates that P3 may be a suitable biomarker for predicting which contacts will lead to motor improvements, while P10 may help predict which contacts lead to SN-related side effects. In the present study, we investigated the correlation between P3 and P10 peak amplitudes and clinical outcomes derived from a classical monopolar review from each electrical contact separately. We then compared our EP biomarker approach to an image-guided programming approach, and finally examined the complementarity of combining both approaches.

## 2. Materials and methods

### 2.1. Participants and surgery

Subjects who met the “UK PD Society Brain Bank Clinical Diagnostic Criteria” for the diagnosis of PD who had DBS surgery at least 3 months prior to enrollment, were included in the study. Directional leads (Vercise Cartesia^®^, Boston Scientific Corporation, Marlborough, MA, USA) (Frey et al., 2022) were bilaterally implanted in the STN. These leads have eight DBS-contacts arranged in a 1-3-3-1 configuration, corresponding to four levels with the middle two levels segmented into three horizontal current steering directions (distal-to-proximal contact numbering of left lead: C1-C8; numbering of right lead: C9-C16, where “C” stands for “Contact”). The surgical procedure was performed with micro-electrode recording technique as standard-of-care, under local anesthesia and intermittent sedation.

The study was approved by the Ethics Committee Research UZ/KU Leuven (S62373) and registered on ClinicalTrials.gov (NCT04658641). All subjects provided oral and written informed consent. The study was conducted in accordance with the Declaration of Helsinki, the Belgian law of May 7th, 2004 on experiments on the human person and in agreement with Good Clinical Practice guidelines.

### 2.2. DBS stimulation during EEG recordings

Subjects were asked to refrain from PD medication intake overnight. One hemisphere was tested at a time, while stimulation in the other hemisphere remained off. Next, the stimulation intensity used for EP recording was defined on the clinical contact configuration (monopolar cathodic pulse with return on the case, frequency of 130 Hz and a pulse width of 60 μs). EPs were recorded at three stimulation intensities: (1) at a subthreshold intensity of 0.5 mA as a positive control where we expect no responses, (2) at the intensity where rigidity was alleviated in the contralateral wrist, (3) at the highest stimulation intensity without non-transient side effects. Due to time constraints, only rigidity was assessed to evaluate clinical effectiveness. After defining the stimulation intensities, the frequency was decreased to 10 Hz. The EPs recorded at all three stimulation intensities were used for an initial intensity analysis (see further). The highest stimulation intensity was then used for all further analyses. Standard-of-care clinical settings are shown in Supplementary Table 1.

### 2.3. EEG recordings and artifact-reduction method

A 64-channel ActiveTwo BioSemi system (Amsterdam, The Netherlands) with a sample rate of 16.384 Hz and a built-in low-pass filter (cut-off frequency of 3,200 Hz) was used for all EEG recordings. This EEG system uses active recording channels positioned according to the internationally standardized 10–20 system (Jasper, 1958) and referenced to the vertex EEG channel (Cz). No additional re-referencing was applied. One additional EEG channel (EXG1) was positioned on the skin over the implantable pulse generator (IPG) to record the stimulation artifact, and served as a trigger channel to align all EPs. The other two additional EEG channels were positioned on the left (EXG2) and right (EXG3) mastoid to record the stimulation artifact at a cranial location with negligible neural responses. We stimulated each of the DBS-contacts individually as well as the segmented contacts in ring mode in randomized order (thus leading to ten tested configurations) for 50 s at 10 Hz, yielding a total of 500 epochs with a duration of 100 ms for each recording. Each epoch was then baseline corrected by subtracting the average of a 1-ms period prior to stimulus onset, after which the epochs were averaged to get the averaged EP. A combination of linear interpolation and template subtraction was applied to reduce the total stimulation-induced artifact. For template subtraction, we generated a scaled template based on the artifact recorded with EEG channels EXG2 and EXG3. After artifact removal, two bandpass 2nd-order Butterworth filters were applied to extract the short- and long-latency EPs: one for evaluation of short-latency EPs (high-pass cutoff frequency: 150 Hz; low-pass cutoff frequency: 1,000 Hz); the other filter was designed for evaluation of long-latency EPs (high-pass cutoff frequency of 1 Hz; low-pass cutoff frequency: 150 Hz). A detailed description of the EEG protocol and template subtraction method for artifact reduction can be found in Chen et al. (2022). Note that the electrophysiological data recorded in some participants included for the current article have already been published in Chen et al. (2022) (see Supplementary Table 2 for details).

### 2.4. Monopolar review assessment

At least 1 month after participating in the EEG recording session, the subjects were asked to come back to the hospital for a double-blinded monopolar review where both the participant and the clinical evaluator were blinded to stimulation intensity and DBS-contact. Stimulation was turned off in both hemispheres at first and then turned on in one DBS-contact (monopolar cathodic pulse with return on the case, frequency of 130 Hz and a pulse width of 60 μs). Stimulation was increased in steps of 0.5 mA and refined in steps of 0.1 mA until rigidity in the contralateral wrist was alleviated. This intensity was termed the bottom of TW (bTW). Stimulation was then again increased in steps of 0.5 mA and refined in 0.1 mA steps until side effects started to appear. This intensity was then termed the top of TW (tTW). TW was defined as the difference between tTW and bTW. One DBS-contact was tested at a time as well as the segmented contacts in ring mode in randomized order. Subjects were asked to refrain from PD medication intake overnight. When side effects were induced before reduction in rigidity was observed, TW was reported as 0 mA.

### 2.5. Imaging data analysis

Lead-DBS, an open-source image processing pipeline (version 2.5.3, Berlin, Germany) (Horn and Kühn, 2015; Horn et al., 2019) was used for postoperative lead reconstruction analysis using the preoperative MRI scan and postoperative CT scan. This analysis allowed for the determination of the specific lead position and orientation on an individual hemispheric level. More specifically, pre-and postoperative images were linearly co-registered and normalized into Montreal Neurological Institute (MNI)-space using the advanced normalization tools module in Lead-DBS (Avants et al., 2008). Electrode trajectories were automatically pre-localized using the PaCER toolbox (Husch et al., 2018) but were manually refined when necessary. Visualization to confirm lead position in reference to relevant anatomical regions was performed in MNI space using the DISTAL atlas (Chakravarty et al., 2006; Ewert et al., 2018), see Supplementary Figure 1 for more details.

### 2.6. Prediction of clinical outcomes using a neuroanatomical sweet spot

One aim was to compare our EP-based approach for predicting clinical outcomes to an established image guided approach. Therefore, we also used a published neuroanatomical sweet spot [motor improvement using part III of the Movement Disorder Society Unified Parkinson’s Disease Rating Scale (UPDRS)] (Dembek et al., 2019) approach to predict clinical outcomes. To accomplish this, we calculated the electric field (EF) at stimulation intensities of 1 mA for each investigated contact using FastField (Baniasadi et al., 2020). We then calculated sweet spot overlap of the EF by multiplying the electric field with the binary mask of the neuroanatomical sweet spot (centers the dorsolateral STN and covered dorsal parts of both sensorimotor STN and associative STN as well as surrounding white matter) (Dembek et al., 2019) and then summed all EF values that laid inside the sweet spot. Since the EF scales linearly with stimulation intensity, one can assume that a contact with larger EF overlap with the neuroanatomical sweet spot at 1 mA would require a lower stimulation intensity to achieve rigidity suppression and thus probably have a lower bTW. Furthermore, electrical stimulation of a contact with a larger EF overlap with the neuroanatomical sweet spot at 1 mA would only provoke side effects at a higher stimulation intensity and thus have a higher tTW.

### 2.7. Software and statistical analysis

All data processing and statistical analyses were done in MATLAB 2022a (Mathworks, Natick, MA, USA). A significance level of 5% was used in all tests. A peak at 3 ms recorded *via* the motor cortex EEG channel ipsilateral to stimulation (i.e., F3 for left and F4 for right hemisphere) was extracted based on the maximum peak amplitude between 2 and 5 ms. Furthermore, a peak at 10 ms recorded *via* the prefrontal EEG channel ipsilateral to stimulation (i.e., AF7 for left and AF8 for right hemisphere) was extracted based on the maximum peak value between 8 and 15 ms. Absolute peak amplitudes (in μV) were used for quantifying the peak amplitudes. According to the central limit theorem, the individual EPs recorded conform to Gaussian assumptions so parametric statistics were used (Central Limit Theorem, 2008). Since each EP consisted of more than 400 epochs, sufficient data was available to perform robust statistics at the individual hemispheric level. In the previous study, a one-way ANOVA was used to investigate if increasing the stimulation intensity significantly affected P3 and P10 amplitude. If no significant effect of intensity was found on the peak amplitude, no further analysis was performed in that hemisphere as we determined that these leads were not close enough to depict a solid P3 or P10 peak. For the remaining hemispheres, we used a linear mixed model to investigate the relationship between the amplitude of P3 or P10 to the monopolar review results at the group level. Linear mixed-effect model analysis was also used to investigate the relationship between the monopolar review results and the predicted clinical outcome measures *via* the sweet spot atlas (Dembek et al., 2019) at the group level. Lastly, we combined both EP and imaging data and calculated additional linear mixed-effect models to investigate the correlation of both EP and imaging data to the monopolar review data. For all linear mixed-effect models, the different hemispheres were included in the model as a random factor (random intercept) and sweet spot EF overlap as well as peak amplitudes and the clinical measures as fixed factors. The distribution of the linear mixed model residuals can be found in Supplementary Figures 2, 3.

For all previously described models, we used the Akaike information criterion (AIC) to investigate the better model fit for predicting monopolar review data. The AIC is a mathematical model to evaluate how well a model fits the data. It is calculated based on the number of independent variables used to build the model and the maximum likelihood estimate of the model. Here, we have calculated three linear mixed models for TW, three for bTW and three for tTW, where we focused on EP amplitudes first, then on image-derived information and lastly on these variables combined. Each of these models generated an AIC value that allows comparison between the three models.

## 3. Results

In total, 10 PD patients participated in this study. Eight patients were tested in one hemisphere while two patients were tested in both hemispheres, yielding a total of 12 hemispheres. Demographics and relevant clinical information are summarized in Table 1. The intensity analysis revealed a significant P3 peak in 9/12 hemispheres and a significant P10 peak in 11/12 hemispheres (see Supplementary Table 2). Short- and long-latency EPs in response to DBS on each individual contact as well as the relationships between detected EPs and therapeutic window measures for all hemispheres separately are shown in Supplementary Figures 4–15.

**TABLE 1:** Demographic data and stimulation parameters.

Subject no.	Gender/age (years)	PD dominant hemicorpus	LEDD (in mg) at time of EEG experiment	Disease duration (in years) at time of EEG experiment	Stimulation intensity (mA) at EEG experiment	Time (in months) between EEG and monopolar review
1R	F/50	R	500	10	6.0	10
1L	F/50	R	500	11	4.0	2
2L	M/55	R	430	9	5.0	18
3L	F/58	L	180	8	3.0	18
4L	F/56	R	430	3	4.0	2
5L	M/71	R	0	9	4.0	6
6L	M/47	L	0	8	6.0	10
7R	F/68	L	0	15	6.0	4
7L	F/68	L	0	15	6.0	4
8R	M/41	L	0	8	6.0	2
9L	F/58	L	320	11	4.8	2
10L	M/59	L	550	15	5.0	2

L, left; R, right; F, female; M, male; LEDD, Levodopa equivalent daily dose.

### 3.1. Relationship between therapeutic window measures and short- and long-latency EPs

**FIGURE 1:**
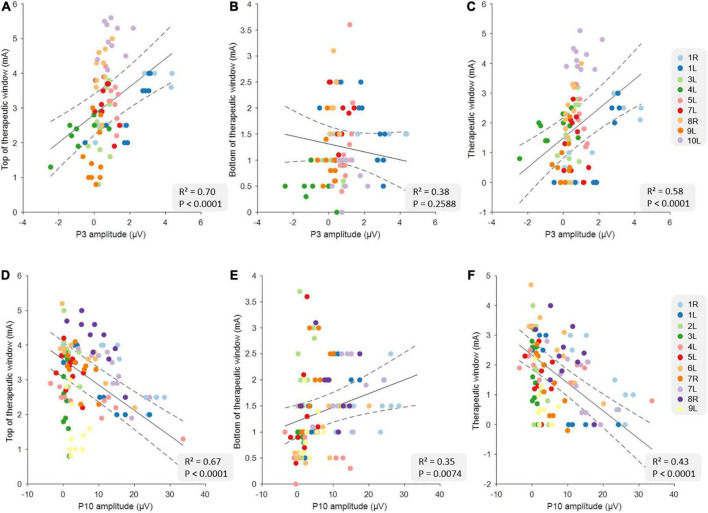
Relationship between clinical measures and the evoked potential (EP) amplitudes at the group level. The top row illustrates the relationships between top of therapeutic window (tTW) **(A)**, bottom of therapeutic window (bTW) **(B),** and therapeutic window (TW) **(C)** and P3 amplitude at the group level (*n* = 90). The bottom row illustrates the relationship between tTW **(D)**, bTW **(E)**, and TW **(F)** and P10 amplitude at the group level (*n* = 110). The colors indicate the 10 deep brain stimulation (DBS)-contacts (i.e., eight individual contacts and two segmented contact levels in ring mode) of the different hemispheres as is shown on the legend on the right side of each row. The black lines indicate the slope of the linear mixed models with CI (dashed lines).

Table 2 shows the full results from six different linear mixed models using P3 and P10 to predict tTW, bTW, and TW. The data from all correlations are shown in Figure 1. In general, we found that the tTW of a DBS-contact could be well approximated using either P3 or P10. Large P3 peak amplitudes corresponded to DBS-contacts showing high tTW intensities (*R*^2^ = 0.70, *p* < 0.0001) while large P10 peak amplitudes corresponded to contacts showing low tTW intensities (*R*^2^ = 0.67, *p* < 0.0001). We observed the opposite relationship between P3 and P10 and bTW: large P10 peak amplitudes corresponded to DBS-contacts showing high bTW intensities (*R*^2^ = 0.35, *p* = 0.0074), while large P3 values corresponded DBS-contacts showing low bTW intensities, but this relationship did not reach significance (*R*^2^ = 0.38, *p* = 0.2588). TW followed a similar pattern to tTW with large P3 peak amplitudes corresponding to DBS-contacts with a wide TW (*R*^2^ = 0.58, *p* < 0.0001) while large P10 peak amplitudes corresponded to contacts with a narrow TW (*R*^2^ = 0.43, *p* < 0.0001).

**TABLE 2:** Linear mixed model statistics.

		tTW	bTW	TW
P3	Equation	tTW ∼ P3 + (1| hemisphere)	bTW ∼ P3 + (1| hemisphere)	TW ∼ P3 + (1| hemisphere)
	AIC	210.06	170.42	259.77
	tStat	4.90	−1.14	4.52
	R^2^	0.70	0.38	0.58
	*p*-value	<0.0001	0.2588	<0.0001
P10	Equation	tTW ∼ P10 + (1| hemisphere)	bTW ∼ P10 + (1| hemisphere)	TW ∼ P10 + (1| hemisphere)
	AIC	228.03	232.52	307.19
	tStat	−7.87	2.73	−6.90
	R^2^	0.67	0.35	0.43
	*p*-value	<0.0001	0.0074	<0.0001
Sweet spot	Equation	tTW ∼ EF overlap + (1| hemisphere)	bTW ∼ EF overlap + (1| hemisphere)	TW ∼ EF overlap + (1| hemisphere)
	AIC	267.71	239.68	335.88
	tStat	6.07	−4.05	7.19
	R^2^	0.68	0.43	0.60
	*p*-value	<0.0001	<0.0001	<0.0001
P3 and sweet spot	Equation	tTW ∼ P3 + EF overlap + (1| hemisphere)	bTW ∼ P3 + EF overlap + (1| hemisphere)	TW ∼ P3 + EF overlap + (1| hemisphere)
	AIC	199.25	163.64	241.5
	tStat	(P3) 3.14 | (EF) 3.73	(P3) 0.23 | (EF) −3.06	(P3) 2.58 | (EF) 4.77
	R^2^	0.75	0.43	0.67
	*p*-value	(P3) 0.0023 | (EF) 0.0003	(P3) 0.8210 | (EF) 0.0029	(P3) 0.0114 | (EF) <0.0001
P10 and sweet spot	Equation	tTW ∼ P10 + EF overlap + (1| hemisphere)	bTW ∼ P10 + EF overlap + (1| hemisphere)	TW ∼ P10 + EF overlap + (1| hemisphere)
	AIC	222.85	227.53	296.37
	tStat	(P10) −4.69 | (EF) 2.77	(P10) 0.56 | (EF) −2.72	(P10) −3.60 | (EF) 3.87
	R^2^	0.70	0.41	0.53
	*p*-value	(P10) <0.0001 | (EF) 0.0067	(P10) 0.5762 | (EF) 0.0076	(P10) 0.0005 | (EF) 0.0002

bTW, bottom of therapeutic window; tTW, top of therapeutic window; TW, therapeutic window; P3, P3 peak; P10, P10 peak.

### 3.2. Relationship between therapeutic window measures and image-derived data

**FIGURE 2:**
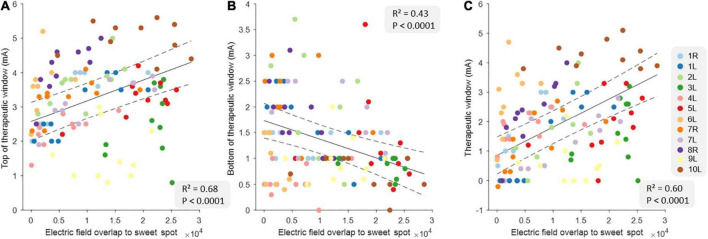
Relationship between clinical measures and electric field overlap to the sweet spot at the group level. The relationship between top of therapeutic window (tTW) **(A)**, bottom of therapeutic window (bTW) **(B)**, and therapeutic window (TW) **(C)** and the electric field overlap to the sweet spot at the group level (*n* = 120). The colors indicate the 10 deep brain stimulation (DBS)-contacts (i.e., eight individual contacts and two segmented contact levels in ring mode) of the different hemispheres as is shown on the legend on the right side. The black lines indicate the slope of the linear mixed models with CI (dashed lines).

Table 2 shows the full results from three different linear models using EF overlap with the sweet spot to predict tTW, bTW, and TW. The data from all correlations are shown in Figure 2. Here, we found that tTW could be estimated using the sweet spot, where a large EF overlap corresponded to DBS-contacts showing high tTW intensities (*R*^2^ = 0.68, *p* < 0.0001). Interestingly, we found that bTW could also be estimated using the sweet spot. However, here a large EF overlap corresponded to contacts where bTW was reached at lower intensities (*R*^2^ = 0.43, *p* < 0.0001). Again, TW followed a similar pattern to tTW with large EF overlap corresponding to DBS-contacts with a wide TW (*R*^2^ = 0.60, *p* < 0.0001).

### 3.3. Relationship between therapeutic window measures and either short-or long-latency EPs and image-derived data

Finally, we investigated the effects of combining either short- or long-latency EPs with the imaging data to predict tTW, bTW, and TW. Table 2 shows the full results from the six different linear models. In general we found that combining either P3 or P10 and imaging data increased the predictive power of the model (i.e., higher R^2^ values and lower AIC values). DBS-contacts with high tTW intensities had large P3 peak amplitudes and large EF overlap (*R*^2^ = 0.75, *p*_P3_ = 0.0023, *p*_EF_ = 0.0003). In contrast, the same high tTW contacts had small P10 peak amplitudes and again large EF overlap (*R*^2^ = 0.70, *p*_P10_ < 0.0001, *p*_EF_ = 0.0067). DBS-contacts with low bTW intensities had large P3 peak amplitudes and large EF overlap (*R*^2^ = 0.43, *p*_P3_ = 0.8210, *p*_EF_ = 0.0029). In contrast, the same low bTW contacts had small P10 peak amplitudes and again large EF overlap (*R*^2^ = 0.41, *p*_P10_ = 0.5762, *p*_EF_ = 0.0076). However, it should be noted that when predicting bTW, neither P3 nor P10 contributed significantly to the combined models. Lastly, DBS-contacts with a wide TW had high P3 peak amplitudes and large EF overlap (*R*^2^ = 0.67, *p*_P3_ = 0.0114, *p*_EF_ < 0.0001), while the same contacts had a low P10 peak amplitude and again large EF overlap (*R*^2^ = 0.53, *p*_P10_ = 0.0005, *p*_EF_ = 0.0002).

**FIGURE 3:**
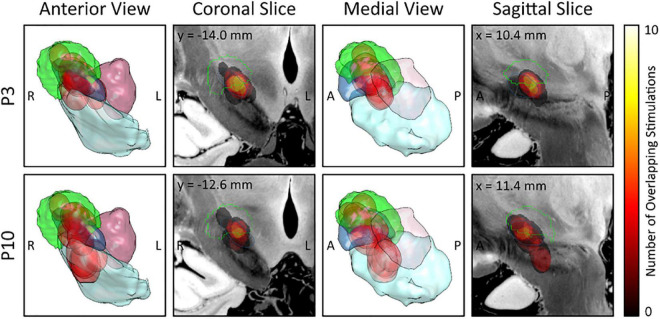
Electric field (EF) overlap to the sweet spot atlas for each deep brain stimulation (DBS)-contact with the highest evoked potential (EP) peak. EF overlap with the sweet spot is shown for each DBS-contact with the highest P3 peak (upper row; *n* = 9) and for each DBS-contact with the highest P10 peak (lower row, *n* = 11). 3D views from anterior (first column) and medial (third column) as well as slices through the weighted-mean-effect image (coronal, second column; sagittal, fourth column) are provided. The Dembek sweet spot (overall motor improvement) is shown in green and the different EFs in red. Anatomic structures: subthalamic nucleus (blue); substantia nigra (mint); red nucleus (lilac). R, right; L, left; A, anterior; P, posterior.

Figure 3 illustrates the EF overlap to the sweet spot for each DBS-contact from each hemisphere where the highest EP peak was recorded, showing that contacts where the strongest P3 peak was recorded, show a large overlap to the sweet spot. Also, contacts where the strongest P10 peak was recorded, overlap largely with the substantia nigra.

## 4. Discussion

The current study demonstrated that EPs can predict therapeutic window outcomes in 10 PD patients (12 hemispheres). In general, the EP morphology was similar to previously published data recorded in similar patient cohorts (Walker et al., 2012; Miocinovic et al., 2018; Peeters et al., 2021). As shown in Supplementary Figure 1, most leads were positioned with the dorsal contacts closer to motor STN thereby leading to a stronger P3 peak in dorsal contacts as was already reported in a previous study (Peeters et al., 2021). Even stimulation from contacts outside of the motor STN’s border resulted in strong P3 peaks, suggesting the involvement of zona incerta (ZI) and white matter tracts such as the hyperdirect pathway (HDP), which are regions also covered by the probabilistic sweet spot (Dembek et al., 2019) from the imaging analysis. Results from this study thus strengthen the hypotheses from previous studies (Butson et al., 2011; Caire et al., 2013) implicating P3 involvement in generating therapeutic DBS effects. Participants where no significant P3 peak was found showed a more medial position of the lead in STN (participants 2L and 6L). For participant 9L, we found no significant P3 peak despite accurate lead positioning, however, the noisy dataset could help explain this.

The ventral contacts were closer to substantia nigra (SN), thereby leading to a stronger P10 peak in ventral contacts, which has already been reported previously (Peeters et al., 2021). The use of an electrophysiological biomarker for side effects to guide programming has been suggested in a previous study by Irwin et al. (2020). For participant 10L, the ventral most contact was positioned within the SN and a P10 peak was expected. Due to the noisy long-latency recordings, however, we decided to exclude this peak for all analyses. The tTW could adequately be predicted by P3 and P10, where contacts with a strong P3 were predictive of a high tTW intensity and contacts with a strong P10 were predictive of a low tTW intensity. Next, the bTW could adequately be predicted by P10, where contacts with a strong P10 were predictive of a high bTW intensity. No significant relationship was observed between P3 peak amplitude and bTW. Lastly, the TW could also be predicted by both P3 and P10 peak amplitudes, where contacts with a strong P3 were predictive of a large TW, while contacts with a strong P10 predicted a narrow TW. These above-described results are in line with the hypothesis that the P3 peak amplitude relates to DBS-contacts that are clinically beneficial, while the P10 peak amplitude relates to side effect-related contacts. We then evaluated if the monopolar review outcomes in our patient cohort could be predicted by images derived information using the Dembek 2019 (Dembek et al., 2019) atlas, which focused on mapping clinical effects (sweet spot) in a similar patient population. We found that contacts with a larger EF overlap to the sweet spot, were predictive of a high tTW intensity, a low bTW intensity and thus, a corresponding wide TW. These analyses confirm that imaging-derived information can be used to guide DBS programming of individual patients, as already shown prior (Maks et al., 2009; Aström et al., 2010; Frankemolle et al., 2010; Mikos et al., 2011; Phibbs et al., 2014; Dembek et al., 2019).

With increasing technological complexity and growing patient populations, an important long-term goal for the DBS field is to optimize programming using data-driven biomarkers approaches. In line with this, we were interested if both electrophysiological data and image-derived information could better predict the clinical measures than each of these datasets separately. We found that the P3 and P10 peaks combined with the EF overlap to the sweet spot could adequately predict the tTW, bTW, and TW. Contacts with a large P3 peak and strong EF overlap were predictive of a high tTW intensity, while contacts with a large P10 peak and weak EF overlap were predictive of a low tTW intensity. Next, contacts with a large P3 peak and strong EF overlap to the sweet spot were predictive of a low bTW, while contacts with a large P10 peak and weak EF overlap were predictive of a high bTW intensity. Note that P3 and P10 did not significantly contribute to these models indicating that these combined models are mainly driven by the EF overlap to the sweet spot. Lastly, contacts with a large P3 peak and strong EF overlap to the sweet spot were predictive of a wide TW, while contacts with a large P10 peak and weak EF overlap were predictive of a narrow TW. Overall, the combination of both electrophysiological and image-derived data predicted the clinical measures better than when each of these factors were added independently (i.e., AIC value decreased which means that adding both features to the model is worth the increased model complexity).

Besides EEG-based approaches to record EP biomarkers, studies have shown that the STN beta-band synchrony can be recorded *via* intraoperative local field potential (LFP) recordings as a correlate of PD symptoms (Kühn et al., 2009). LFP-based programming is becoming a promising tool for advancing DBS therapy toward a more objective and adaptive manner (Meidahl et al., 2017; Noor and McIntyre, 2021; Chen et al., 2022; Darcy et al., 2022; Hirschmann et al., 2022; Shah et al., 2022). A distinct advantage of cortical EPs over LFPs recorded from the lead, is that they can give much more information on the specific neural networks being activated when different DBS contacts are stimulated since both deep and cortical sources can be recorded using EEG (Gransier et al., 2021a,b). We furthermore believe that an EEG session is less burdensome on the patients as it does not require any interaction of the patient whilst still leading to more objective data compared to a monopolar review assessment. However, completing a whole session can take up to 2 h, thereby still requesting some time and effort from the patient. Other emerging tools to guide programming are fMRI combined with machine learning (Phibbs et al., 2014) and biophysical model-based programming (Howell et al., 2021). Furthermore, a recent study (Roediger et al., 2022) even developed a fully automated algorithm to help guide the programming of individual patients, termed “Stimfit.” Adding electrophysiological data to image-derived information could improve the programming of individual patients in a complementary manner to optimize DBS programming. Also, image-based programming with commercial software has also proven an effective programming tool that leads to non-inferior motor symptom control compared to standard programming (Ewert et al., 2018). fMRI acquisition has been investigated as a means to predict optimal DBS stimulation parameters to enhance the therapeutic potential of DBS (Boutet et al., 2021). Recently, a study was published where the use of fMRI in combination with machine learning led to reproducible functional brain activity maps of therapeutic DBS activity in a PD patient cohort, stating that fMRI may be used to facilitate individualized programming and may guide DBS programming.

There are some potential limitations to be noted. The sample size is modest but note that programming patients, lead positioning and monopolar review performance all happened on a patient-specific level. Also, we were unable to record a P3 or P10 peak in 25 and 8% of the tested hemispheres, respectively, most likely due to lead location Also, we only considered rigidity as a clinical outcome measure when performing the monopolar reviews. However, the sweet spot atlas (Dembek et al., 2019) focused on overall motor improvement using part III of the UPDRS, thereby assessing more clinical outcomes than just rigidity. Thus, P3 may be more closely linked to other clinical effects but further investigation is needed to confirm this. This limitation may account for the non-significant outcomes in the model investigating the relationship between bTW and P3. Furthermore, given the stimulation intensity, it may be that the internal capsule also contributes to P3, meaning that P3 may also reflect capsular side effects.

In conclusion, we found that both the TW and tTW measured from the different DBS-contact can be predicted based on electrophysiological data and based on image-derived information separately. However, combining both electrophysiological and image-derived data in one linear mixed model further improves the prediction of clinical outcomes. Ultimately, these EPs may serve as biomarkers to guide programming as a complementary approach to image-guided programming of individual DBS patients in a more data-driven manner.

## Data availability statement

The raw data supporting the conclusions of this article will be made available by the authors, without undue reservation.

## Ethics statement

The studies involving human participants were reviewed and approved by the Ethics Committee Research UZ/KU Leuven. The patients/participants provided their written informed consent to participate in this study.

## Author contributions

JP, AB, TV, RG, BN, and MM contributed to the conception and design of the study. JP, TD, and MM performed the statistical analysis. JP wrote the first draft of the manuscript. All authors contributed to manuscript revision, read and approved the submitted version.
