# Supplementary material for: Towards biomarker-based optimization of deep brain stimulation in Parkinson’s disease patients
Source: Front Neurosci. 2023 Jan 11;16:1091781. doi: 10.3389/fnins.2022.1091781 (PMC9875598; doi:10.3389/fnins.2022.1091781)
Supplement: Supplementary file 1 [file Data_Sheet_1.pdf]

Supplementary Material

1 Visualization of the lead to confirm lead position in reference to relevant anatomical regions

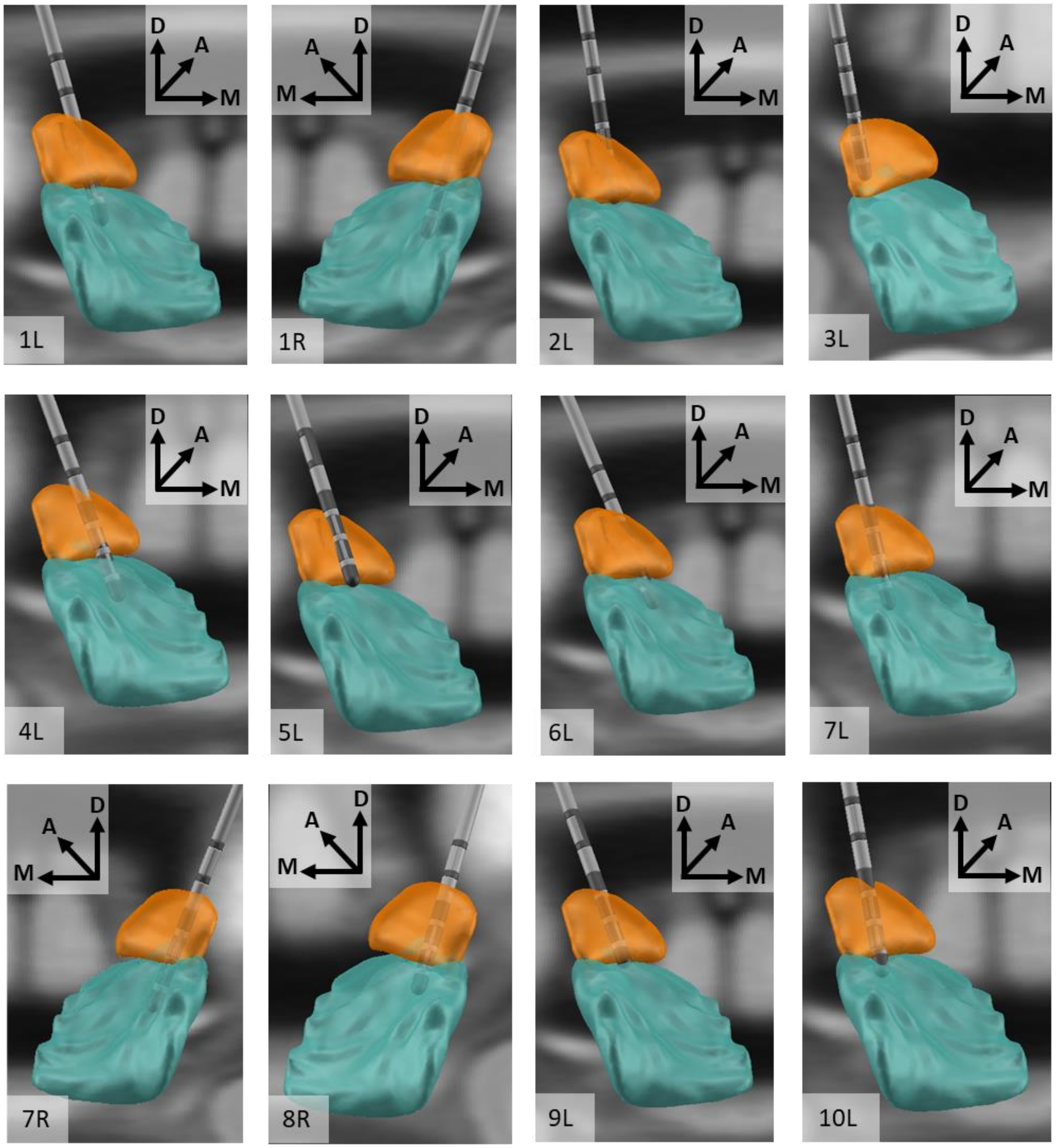

Supplementary figure 1: Lead-DBS reconstruction of lead position

Each panel shows the reconstruction of the DBS-lead in reference to the subthalamic nucleus (orange) and the substantia nigra (blue) for each participant. Legend: D: dorsal, A: anterior, M: medial.

2 Distribution of linear mixed model residuals

Supplementary figure 2 shows the distribution of the residuals from the linear mixed models. The upper row shows the distribution of the residuals calculated from the linear mixed models investigating the relationship between tTW and P3 (left), between bTW and P3 (middle) and between TW and P3 (right). The middle row shows the distribution of the residuals calculated from the linear mixed models investigating the relationship between tTW and P10 (left), between bTW and P10 (middle) and between TW and P10 (right). The lower row shows the distribution of the residuals calculated from the linear mixed models investigating the relationship between tTW and the overlap to the sweet spot (left), between bTW and the overlap to the sweet spot (middle) and between TW and the overlap to the sweet spot (right).

Supplementary figure 3 shows the distribution of the residuals from the combined linear mixed models. The upper row shows the distribution of the residuals calculated from the linear mixed models investigating the relationship between tTW, P3 and the overlap to the sweet spot (left), between bTW, P3 and the overlap to the sweet spot (middle) and between TW, P3 and the overlap to the sweet spot (right). The bottom row shows shows the distribution of the residuals calculated from the linear mixed models investigating the relationship between tTW, P10 and the overlap to the sweet spot (left), between bTW, P10 and the overlap to the sweet spot (middle) and between TW, P10 and the overlap to the sweet spot (right).

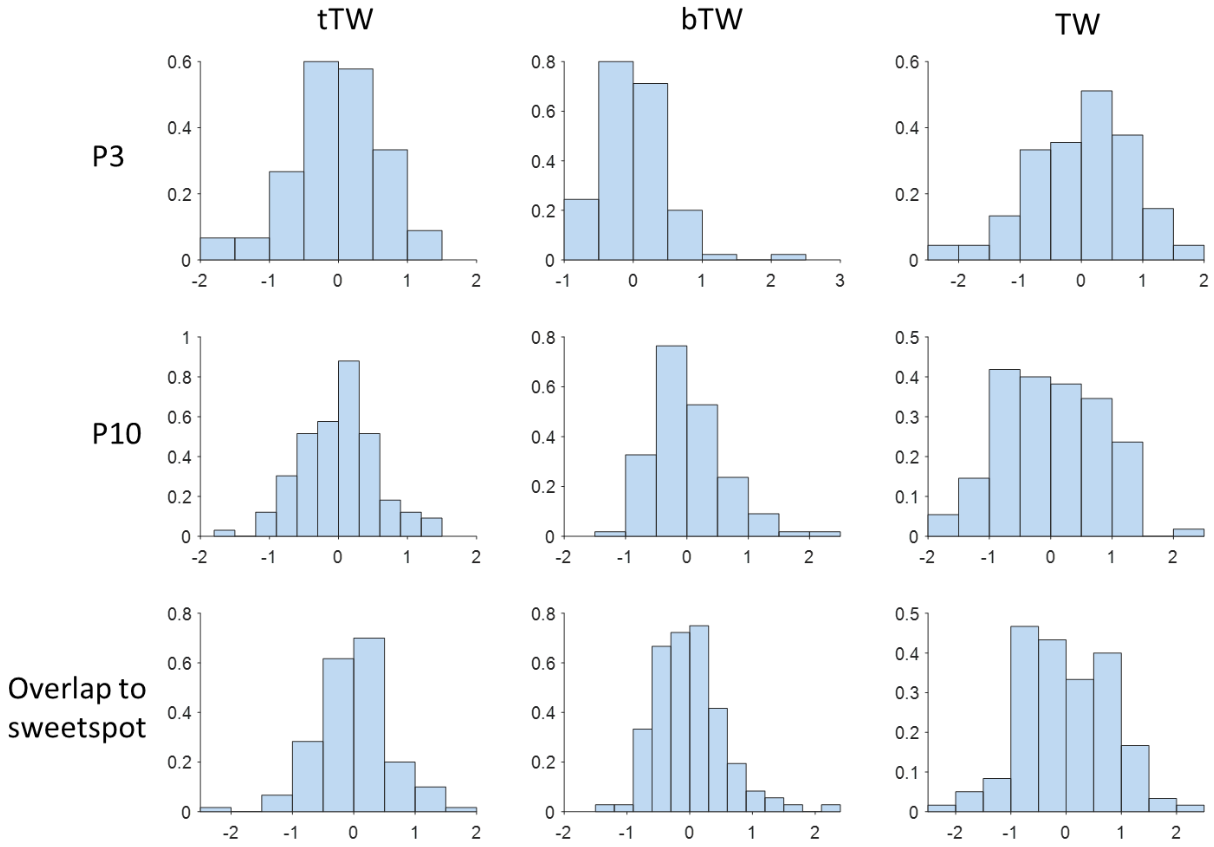

Supplementary figure 2: distribution of linear mixed models residuals

The upper row shows the distribution of the residuals calculated from the linear mixed models investigating the relationship between tTW and P3 (left), between bTW and P3 (middle) and between TW and P3 (right). The middle row shows the distribution of the residuals calculated from the linear mixed models investigating the relationship between tTW and P10 (left), between bTW and P10 (middle) and between TW and P10 (right). The lower row shows the distribution of the residuals calculated from the linear mixed models investigating the relationship between tTW and the overlap to the sweet spot (left), between bTW and the overlap to the sweet spot (middle) and between TW and the overlap to the sweet spot (right).

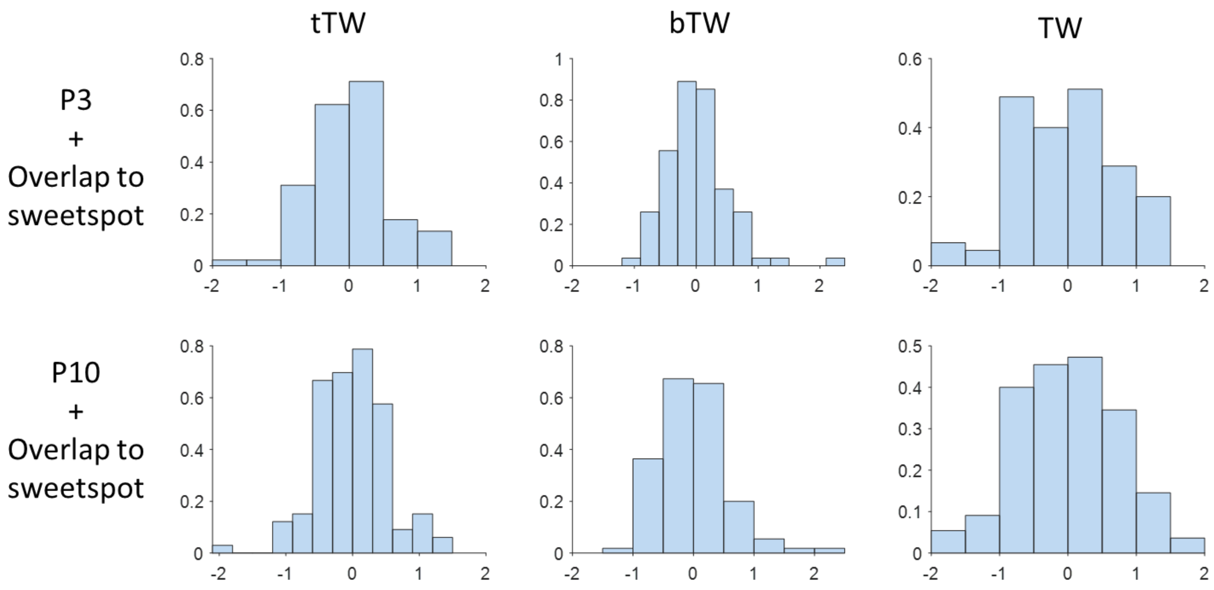

Supplementary figure 3: distribution of the combined linear mixed model residuals

The upper row shows the distribution of the residuals calculated from the linear mixed models investigating the relationship between tTW, P3 and the overlap to the sweet spot (left), between bTW, P3 and the overlap to the sweet spot (middle) and between TW, P3 and the overlap to the sweet spot (right). The bottom row shows shows the distribution of the residuals calculated from the linear mixed models investigating the relationship between tTW, P10 and the overlap to the sweet spot (left), between bTW, P10 and the overlap to the sweet spot (middle) and between TW, P10 and the overlap to the sweet spot (right).

3 Standard-of-care clinical settings

Subject No.	Active contacts	Type of stimulation	Stimulation intensity (mA)	Pulse width (µs)	Frequency (Hz)
1R	6-(10%)<br>8-(90%)	Monopolar	2.6	60	139
1L	5-(4%)<br>6-(32%)<br>7-(4%)<br>8-(20%)	Monopolar	4.6	60	130
2L	8-(100%)	Monopolar	2.8	60	130
3L	5-(40%)<br>6-(40%)<br>8-(80%)	Monopolar	1.6	60	130
4L	2-(34%)<br>3-(33%)<br>4-(33%)	Monopolar	1.7	60	130
5L	5-(34%)<br>6-(33%)<br>7-(33%)	Monopolar	3.0	60	130
6L	5-(20%)<br>7-(60%)<br>8-(20%)	Monopolar	3.2	60	130
7R	6-(30%)<br>8-(70%)	Monopolar	4.6	50	104
7L	5-(13%)<br>6-(54%)<br>7-(13%)<br>8-(20%)	Monopolar	2.7	60	104
8R	5+(70%)<br>6+(30%)<br>8-(100%)	Bipolar	4.4	110	130
9L	5-(50%)<br>8-(80%)	Monopolar	1.7	60	130
10L	2-(34%)<br>3-(33%)<br>4-(33%)	Monopolar	2.6	60	128

Supplementary table 1: Standard-of-care clinical settings of all participants. The percentage after the contact used for stimulation represents the percentage of the total current.

Legend: right hemisphere, L: left hemisphere, (-): cathodic stimulation, (+): anodic stimulation

4 Intensity analysis to investigate if increasing stimulation intensity significantly increases EP amplitude

The short-latency EPs were recorded via the motor cortex EEG channel ipsilateral to stimulation (i.e., EEG channel F3 for the left and F4 for the right hemisphere). The long-latency EPs were recorded via the prefrontal cortex EEG channel ipsilateral to stimulation (i.e., EEG channel AF7 for the left and AF8 for the right hemisphere). Based on the analysis of the previous study by Peeters et al., we found a significant P3 peak in nine hemispheres and a significant P10 peak in eleven hemispheres. Supplementary table 1 shows a recap of this analysis.

Subject No.	Subject No. in previous study[2]	Intensity P-value (F-statistics)	
		P3	P10
1L	1L	< 0.0001 (257.8)	< 0.0001 (770.9)
1R	1R	< 0.0001 (22.2)	< 0.0001 (862.2)
2L	2L	NS (1.48)	< 0.0001 (34.5)
3L	3L	0.0184 (4.0)	0.0268 (3.6)
4L	4L	0.0147 (4.2)	< 0.0001 (285.5)
5L	5L	0.0002 (8.6)	< 0.0001 (11.8)
6L	6L	NS (0.12)	< 0.0001 (267.1)
7L	7L	< 0.0001 (22.7)	< 0.0001 (143.5)
7R	7R	NS (0.4)	0.0419 (3.2)
8R	8R	< 0.0001 (35.9)	< 0.0001 (123.4)
9L	-	0.0004 (7.9)	< 0.0001 (51.9)
10L	-	<0.0001 (11.8)	NS (2.7)
Total (%)	-	9/12 (75%)	11/12 (92%)

Supplementary table 2: Recap of ANOVA results investigating the effect of stimulation intensity on P3 and P10 peak amplitude

Legend: R: right hemisphere, L: left hemisphere

5 Relationship between clinical measures and short- and long-latency EPs – patient level

Supplementary figures 4-15 show the short (A) and long-latency (B) EPs recorded in response to DBS on each individual contact for every hemisphere tested. A linear model was used to investigate the relationship between TW and the P3 peak amplitude, between bTW and the P3 peak amplitude and lastly between tTW and the P10 peak amplitude. Results of these linear models are shown in supplementary table 2. We observed a significant correlation between P3 and tTW in 4/9 hemispheres, between P3 and bTW in 1/9 hemispheres and between P3 and TW in 3/9 hemispheres. Furthermore, we observed a significant correlation between P10 and tTW in 6/11 hemispheres, between P10 and bTW in 3/11 hemispheres and between P10 and TW in 6/11 hemispheres. The exact R^2 and p-values are shown in the graphs.

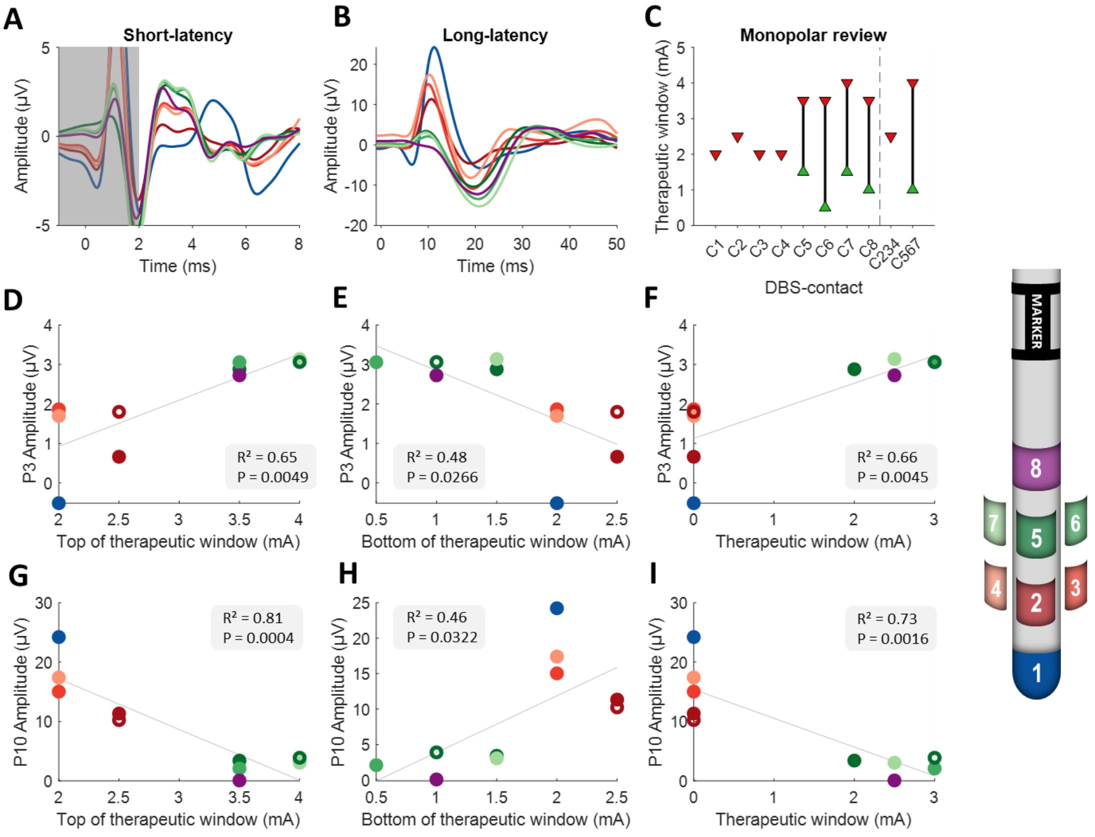

Supplementary figure 4: Short- and long-latency EPs are correlated with clinical measures – participant 1 left hemi

Short- (A) and long-latency (B) EPs recorded from each DBS-contact. Each EP is colored differently, corresponding to the colors of the electrode on the right side. The grey transparent box indicates the time window (-1 to 2 ms) where residual artifact might still be present. (C) shows the results of the monopolar review. The green triangles represent bTW while the red triangles indicate tTW for each DBS-contact separately, as well as the segmented contacts in ring mode. The middle panels show the correlation between P3 amplitude and tTW (D), between P3 amplitude and bTW (E) and between P3 amplitude and tTW (F). The lower panels how the correlation between P10 amplitude and tTW (G), between P10 amplitude and bTW (H) and between P10 amplitude and tTW (I). The different colors represent the data recorded from each different DBS-contact as is indicated on the electrode on the right. The full dots show the separate DBS-contacts while the open circles represent the segmented contacts in ring mode (red circle: C2-3-4; green circle: C5-6-7).

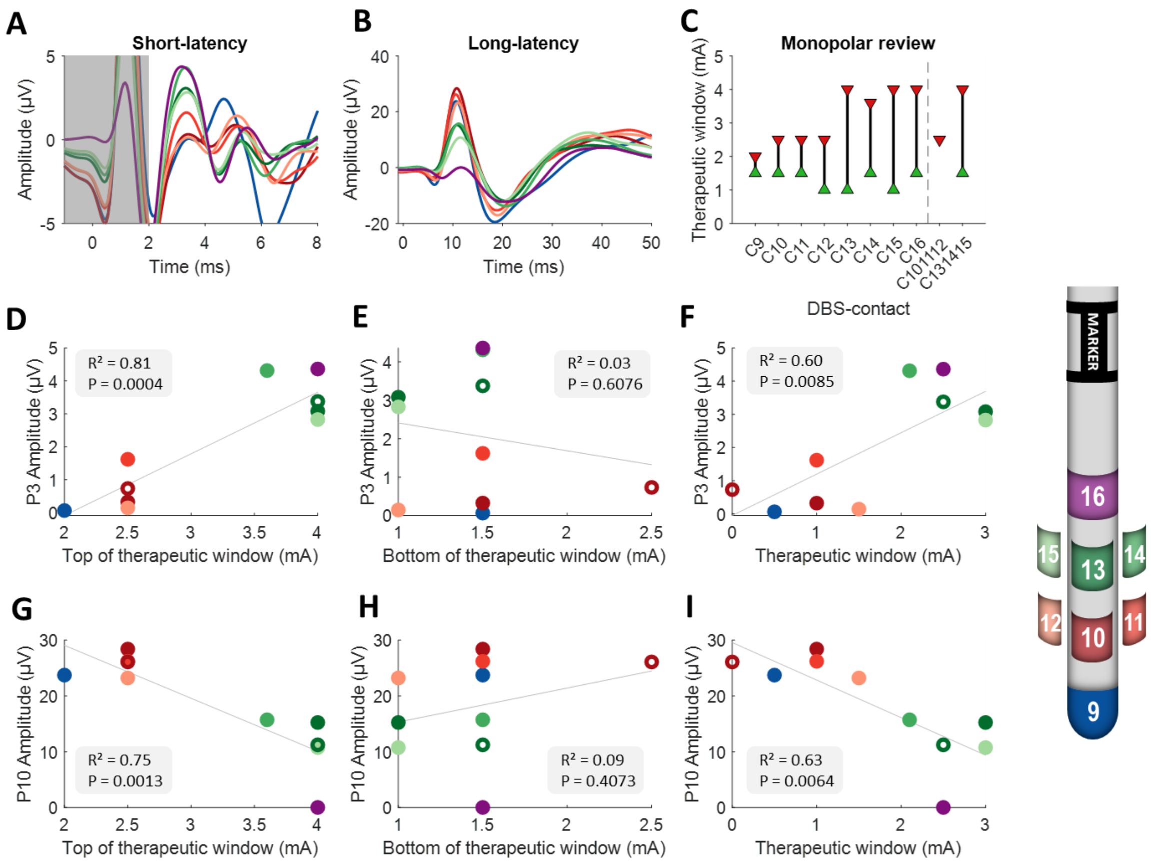

Supplementary figure 5: Short- and long-latency EPs are correlated with clinical measures – participant 1 right hemi

Short- (A) and long-latency (B) EPs recorded from each DBS-contact. Each EP is colored differently, corresponding to the colors of the electrode on the right side. The grey transparent box indicates the time window (-1 to 2 ms) where residual artifact might still be present. (C) shows the results of the monopolar review. The green triangles represent bTW while the red triangles indicate tTW for each DBS-contact separately, as well as the segmented contacts in ring mode The middle panels show the correlation between P3 amplitude and tTW (D), between P3 amplitude and bTW (E) and between P3 amplitude and tTW (F). The lower panels how the correlation between P10 amplitude and tTW (G), between P10 amplitude and bTW (H) and between P10 amplitude and tTW (I). The different colors represent the data recorded from each different DBS-contact as is indicated on the electrode on the right. The full dots show the separate DBS-contacts while the open circles represent the segmented contacts in ring mode (red circle: C10-11-12; green circle: C13-14-15).

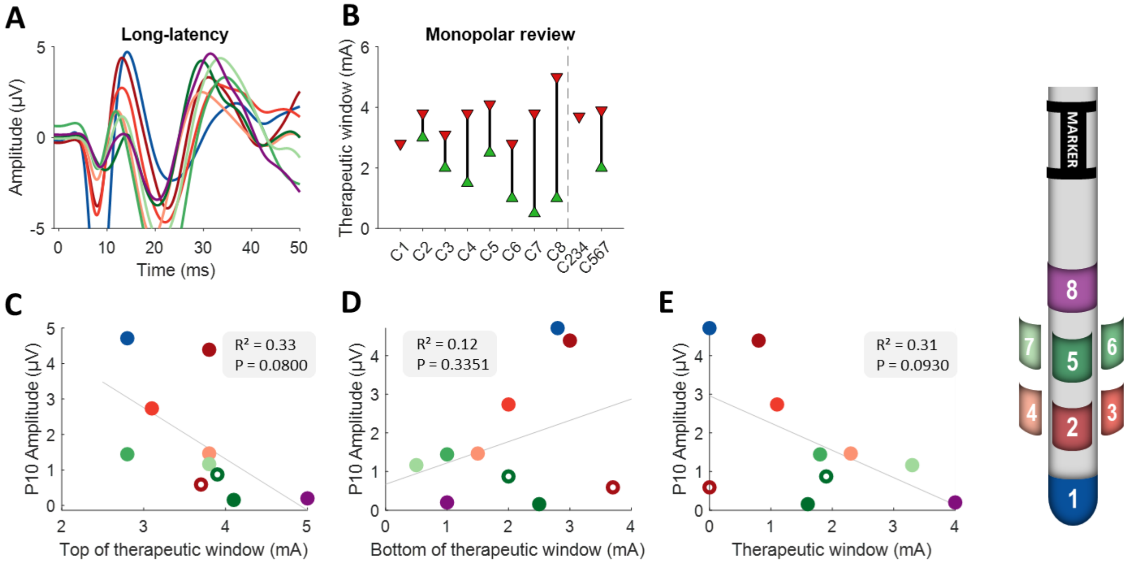

Supplementary figure 6: Short- and long-latency EPs are correlated with clinical measures – participant 2 left hemi

Long-latency (A) EPs recorded from each DBS-contact. Each EP is colored differently, corresponding to the colors of the electrode on the right side. The grey transparent box indicates the time window (-1 to 2 ms) where residual artifact might still be present. (B) shows the results of the monopolar review. The green triangles represent bTW while the red triangles indicate tTW for each DBS-contact separately, as well as the segmented contacts in ring mode. The lower panels show the correlation between P3 amplitude and tTW (C), between P3 amplitude and bTW (D) and between P3 amplitude and tTW (E). The different colors represent the data recorded from each different DBS-contact as is indicated on the electrode on the right. The full dots show the separate DBS-contacts while the open circles represent the segmented contacts in ring mode (red circle: C2-3-4; green circle: C5-6-7).

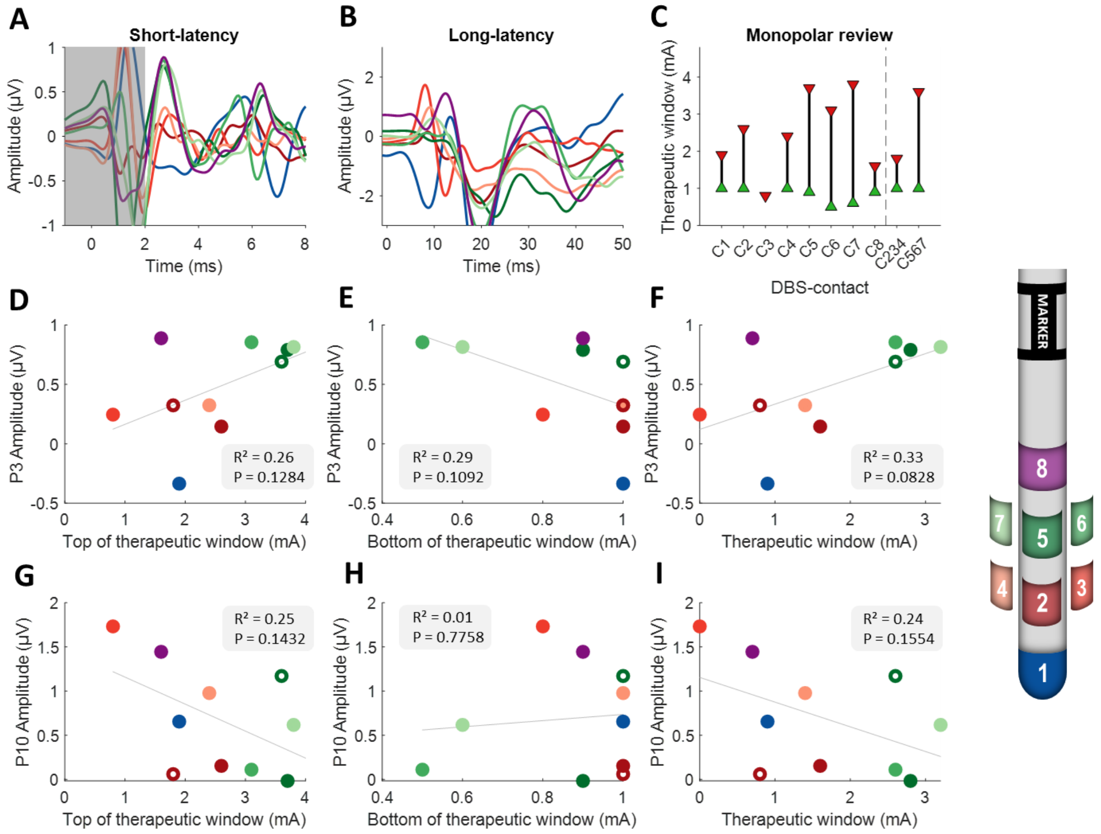

Supplementary figure 7: Short- and long-latency EPs are correlated with clinical measures – participant 3 left hemi

Short- (A) and long-latency (B) EPs recorded from each DBS-contact. Each EP is colored differently, corresponding to the colors of the electrode on the right side. The grey transparent box indicates the time window (-1 to 2 ms) where residual artifact might still be present. (C) shows the results of the monopolar review. The green triangles represent bTW while the red triangles indicate tTW for each DBS-contact separately, as well as the segmented contacts in ring mode. The middle panels show the correlation between P3 amplitude and tTW (D), between P3 amplitude and bTW (E) and between P3 amplitude and tTW (F). The lower panels how the correlation between P10 amplitude and tTW (G), between P10 amplitude and bTW (H) and between P10 amplitude and tTW (I). The different colors represent the data recorded from each different DBS-contact as is indicated on the electrode on the right. The full dots show the separate DBS-contacts while the open circles represent the segmented contacts in ring mode (red circle: C2-3-4; green circle: C5-6-7).

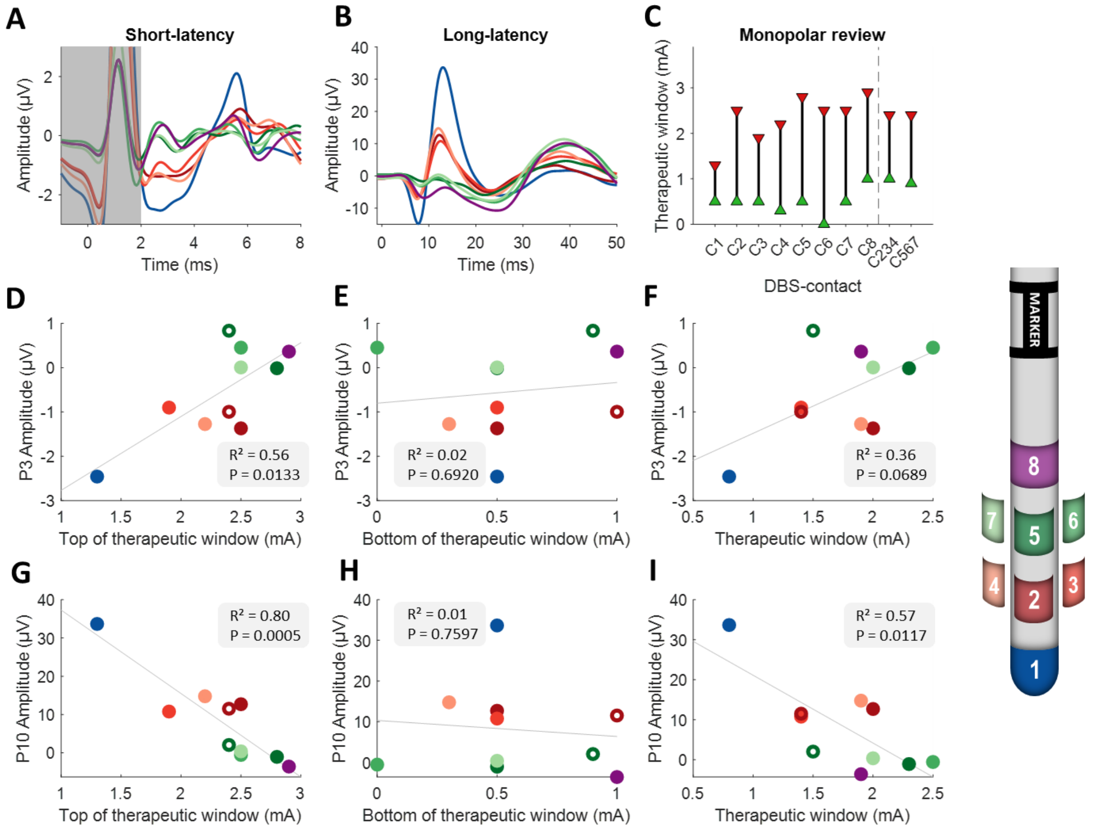

Supplementary figure 8: Short- and long-latency EPs are correlated with clinical measures – participant 4 left hemi

Short- (A) and long-latency (B) EPs recorded from each DBS-contact. Each EP is colored differently, corresponding to the colors of the electrode on the right side. The grey transparent box indicates the time window (-1 to 2 ms) where residual artifact might still be present. (C) shows the results of the monopolar review. The green triangles represent bTW while the red triangles indicate tTW for each DBS-contact separately, as well as the segmented contacts in ring mode. The middle panels show the correlation between P3 amplitude and tTW (D), between P3 amplitude and bTW (E) and between P3 amplitude and tTW (F). The lower panels how the correlation between P10 amplitude and tTW (G), between P10 amplitude and bTW (H) and between P10 amplitude and tTW (I). The different colors represent the data recorded from each different DBS-contact as is indicated on the electrode on the right. The full dots show the separate DBS-contacts while the open circles represent the segmented contacts in ring mode (red circle: C2-3-4; green circle: C5-6-7).

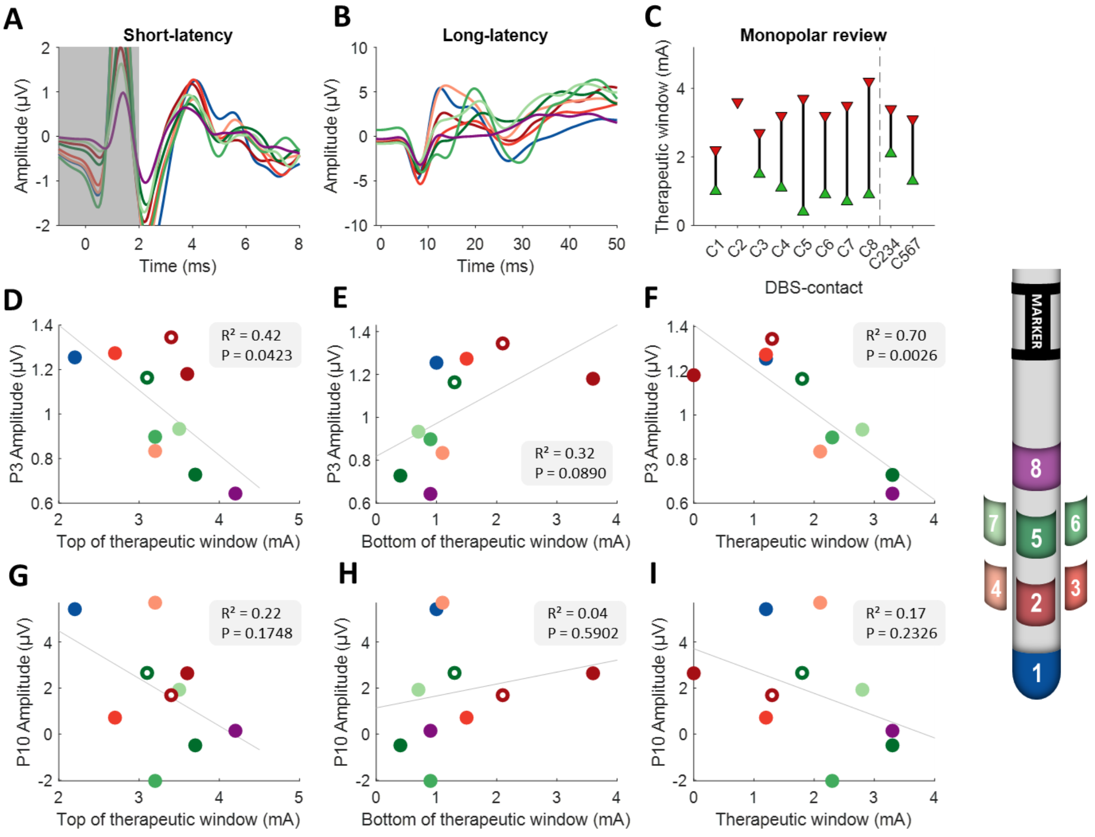

Supplementary figure 9: Short- and long-latency EPs are correlated with clinical measures – participant 5 left hemi

Short- (A) and long-latency (B) EPs recorded from each DBS-contact. Each EP is colored differently, corresponding to the colors of the electrode on the right side. The grey transparent box indicates the time window (-1 to 2 ms) where residual artifact might still be present. (C) shows the results of the monopolar review. The green triangles represent bTW while the red triangles indicate tTW for each DBS-contact separately, as well as the segmented contacts in ring mode The middle panels show the correlation between P3 amplitude and tTW (D), between P3 amplitude and bTW (E) and between P3 amplitude and tTW (F). The lower panels how the correlation between P10 amplitude and tTW (G), between P10 amplitude and bTW (H) and between P10 amplitude and tTW (I). The different colors represent the data recorded from each different DBS-contact as is indicated on the electrode on the right. The full dots show the separate DBS-contacts while the open circles represent the segmented contacts in ring mode (red circle: C2-3-4; green circle: C5-6-7).

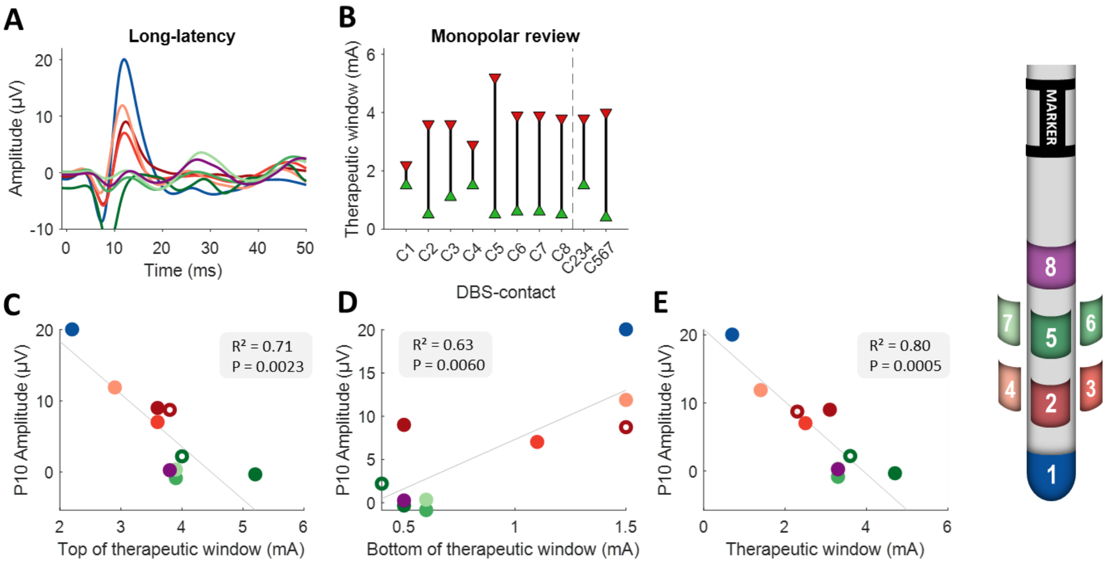

Supplementary figure 10: Short- and long-latency EPs are correlated with clinical measures – participant 6 left hemi

Long-latency (A) EPs recorded from each DBS-contact. Each EP is colored differently, corresponding to the colors of the electrode on the right side. The grey transparent box indicates the time window (-1 to 2 ms) where residual artifact might still be present. (B) shows the results of the monopolar review. The green triangles represent bTW while the red triangles indicate tTW for each DBS-contact separately, as well as the segmented contacts in ring mode. The lower panels show the correlation between P3 amplitude and tTW (C), between P3 amplitude and bTW (D) and between P3 amplitude and tTW (E). The different colors represent the data recorded from each different DBS-contact as is indicated on the electrode on the right. The full dots show the separate DBS-contacts while the open circles represent the segmented contacts in ring mode (red circle: C2-3-4; green circle: C5-6-7).

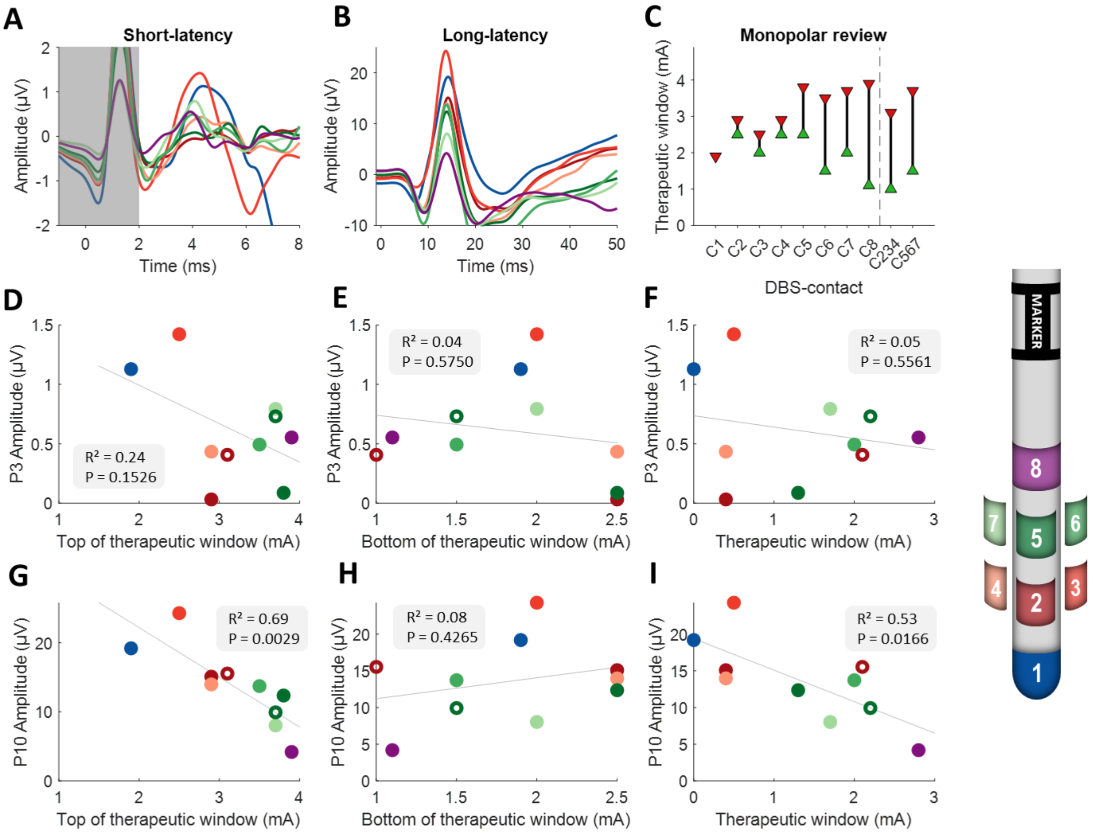

Supplementary figure 11: Short- and long-latency EPs are correlated with clinical measures – participant 7 left hemi

Short- (A) and long-latency (B) EPs recorded from each DBS-contact. Each EP is colored differently, corresponding to the colors of the electrode on the right side. The grey transparent box indicates the time window (-1 to 2 ms) where residual artifact might still be present. (C) shows the results of the monopolar review. The green triangles represent bTW while the red triangles indicate tTW for each DBS-contact separately, as well as the segmented contacts in ring mode The middle panels show the correlation between P3 amplitude and tTW (D), between P3 amplitude and bTW (E) and between P3 amplitude and tTW (F). The lower panels how the correlation between P10 amplitude and tTW (G), between P10 amplitude and bTW (H) and between P10 amplitude and tTW (I). The different colors represent the data recorded from each different DBS-contact as is indicated on the electrode on the right. The full dots show the separate DBS-contacts while the open circles represent the segmented contacts in ring mode (red circle: C2-3-4; green circle: C5-6-7).

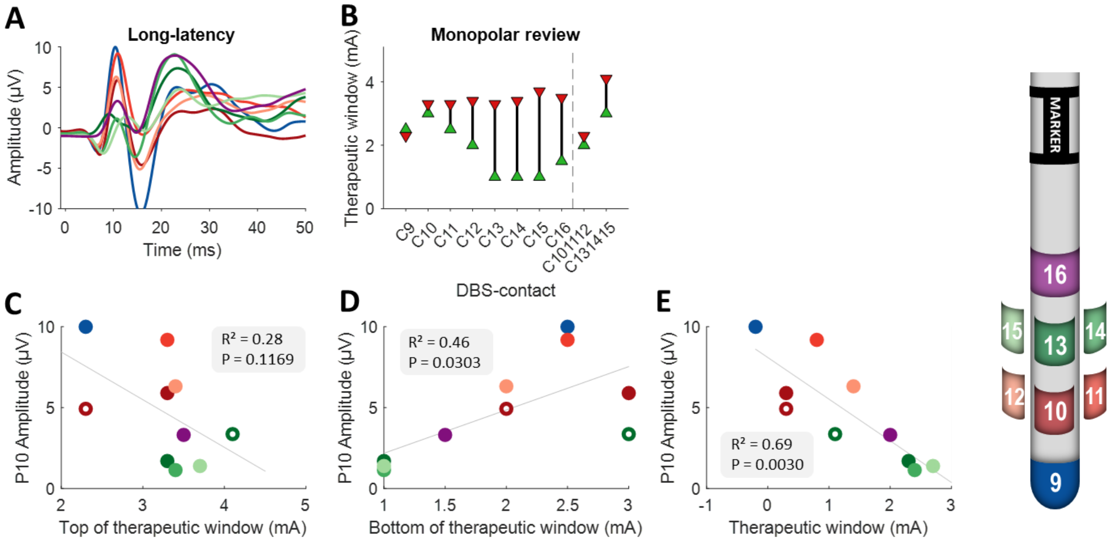

Supplementary figure 22: Short- and long-latency EPs are correlated with clinical measures – participant 7 right hemi

Long-latency (A) EPs recorded from each DBS-contact. Each EP is colored differently, corresponding to the colors of the electrode on the right side. The grey transparent box indicates the time window (-1 to 2 ms) where residual artifact might still be present. (B) shows the results of the monopolar review. The green triangles represent bTW while the red triangles indicate tTW for each DBS-contact separately, as well as the segmented contacts in ring mode The lower panels show the correlation between P3 amplitude and tTW (C), between P3 amplitude and bTW (D) and between P3 amplitude and tTW (E). The different colors represent the data recorded from each different DBS-contact as is indicated on the electrode on the right. The full dots show the separate DBS-contacts while the open circles represent the segmented contacts in ring mode (red circle: C10-11-12; green circle: C13-14-15).

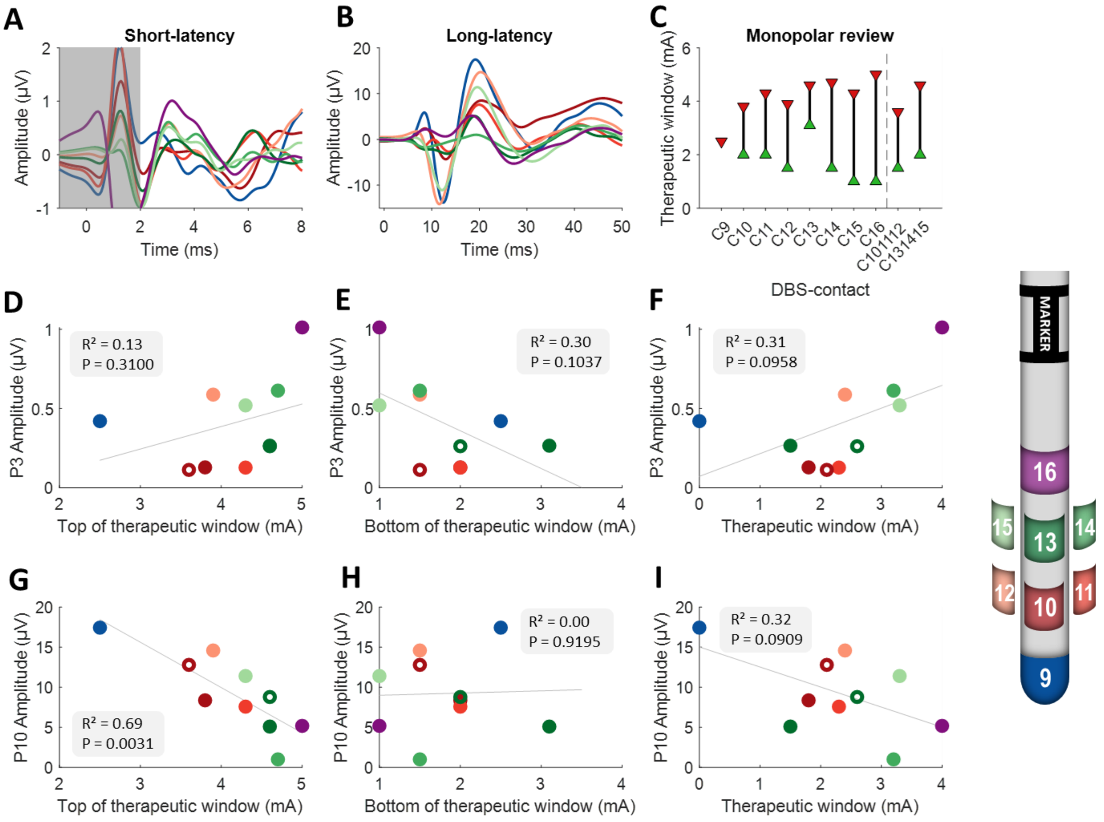

Supplementary figure 33: Short- and long-latency EPs are correlated with clinical measures – participant 7 right hemi

Short- (A) and long-latency (B) EPs recorded from each DBS-contact. Each EP is colored differently, corresponding to the colors of the electrode on the right side. The grey transparent box indicates the time window (-1 to 2 ms) where residual artifact might still be present. (C) shows the results of the monopolar review. The green triangles represent bTW while the red triangles indicate tTW for each DBS-contact separately, as well as the segmented contacts in ring mode The middle panels show the correlation between P3 amplitude and tTW (D), between P3 amplitude and bTW (E) and between P3 amplitude and tTW (F). The lower panels how the correlation between P10 amplitude and tTW (G), between P10 amplitude and bTW (H) and between P10 amplitude and tTW (I). The different colors represent the data recorded from each different DBS-contact as is indicated on the electrode on the right. The full dots show the separate DBS-contacts while the open circles represent the segmented contacts in ring mode (red circle: C10-11-12; green circle: C13-14-15).

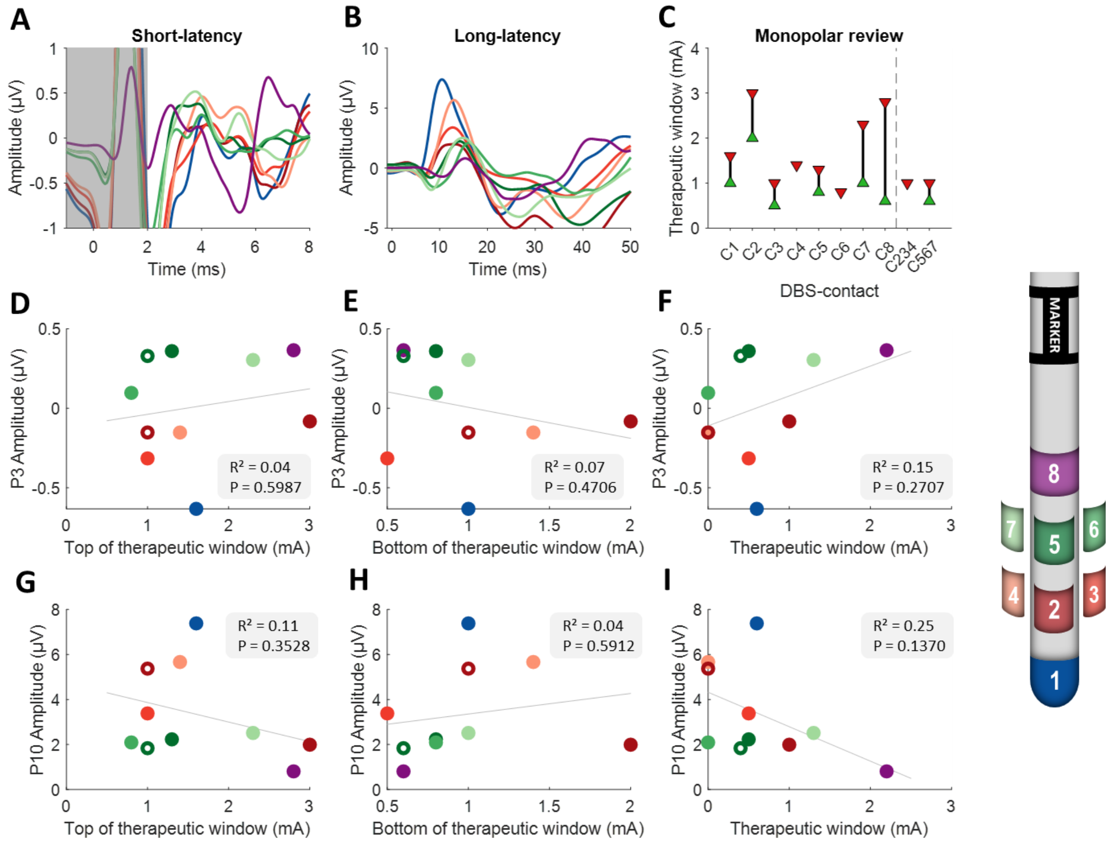

Supplementary figure 44: Short- and long-latency EPs are correlated with clinical measures – participant 9 left hemi

Short- (A) and long-latency (B) EPs recorded from each DBS-contact. Each EP is colored differently, corresponding to the colors of the electrode on the right side. The grey transparent box indicates the time window (-1 to 2 ms) where residual artifact might still be present. (C) shows the results of the monopolar review. The green triangles represent bTW while the red triangles indicate tTW for each DBS-contact separately, as well as the segmented contacts in ring mode. The middle panels show the correlation between P3 amplitude and tTW (D), between P3 amplitude and bTW (E) and between P3 amplitude and tTW (F). The lower panels how the correlation between P10 amplitude and tTW (G), between P10 amplitude and bTW (H) and between P10 amplitude and tTW (I). The different colors represent the data recorded from each different DBS-contact as is indicated on the electrode on the right. The full dots show the separate DBS-contacts while the open circles represent the segmented contacts in ring mode (red circle: C2-3-4; green circle: C5-6-7).

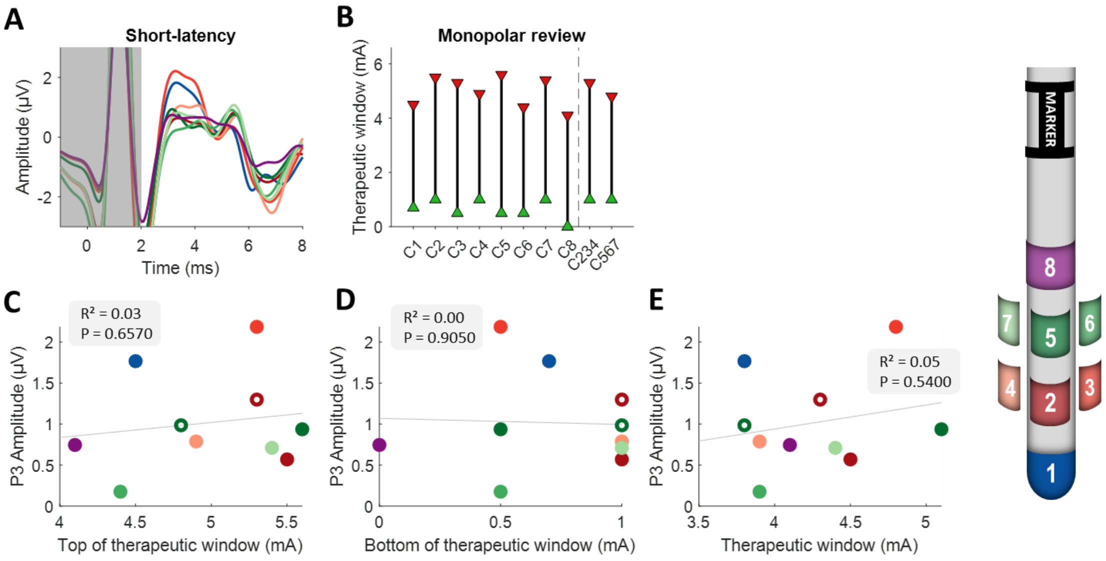

Supplementary figure 55: Short-latency EPs are correlated with clinical measures – participant 10 left hemi

Short-latency (A) EPs recorded from each DBS-contact. Each EP is colored differently, corresponding to the colors of the electrode on the right side. (B) shows the results of the monopolar review. The green triangles represent bTW while the red triangles indicate tTW for each DBS-contact separately, as well as the segmented contacts in ring mode. The lower panels show the correlation between P3 amplitude and tTW (C), between P3 amplitude and bTW (D) and between P3 amplitude and tTW (E). The different colors represent the data recorded from each different DBS-contact as is indicated on the electrode on the right. The full dots show the separate DBS-contacts while the open circles represent the segmented contacts in ring mode (red circle: C2-3-4; green circle: C5-6-7).
